# Facts Worth Knowing

**Published:** 1883-01

**Authors:** 


### FACTS WORTH KNOWING.

That salt fish are quickest and best freshened by soaking in sour milk.

That cold rain water and soap will remove machine grease from washable fabrics.

That fish may be scaled much easier by first dipping them into boiling water for a minute.

That fresh meat, beginning to sour, will sweeten if placed out of doors in the cool air over night.

That milk which has changed may be sweetened or rendered fit for use again by stirring in a little soda.

That boiling starch is much improved by the addition of sperm, or salt, or both, or a little gum arabic, dissolved.

That a tablespoonful of turpentine, boiled with your white clothes, will greatly aid the whitening process.

That kerosene will soften boots and shoes that have been hardened by water, and will render them pliable as new.

That clear boiling water will remove tea stains ; pour the water through the stain, and thus prevent its spreading over the fabric.

That salt will curdle new milk, hence, in preparing milk porridge, gravies, etc., the salt should not be added until the dish is prepared.

That kerosene will make your tea-kettle as bright as new. Saturate a woolen rag and rub with it. It will also remove stains from the clean varnished furniture.

That blue ointment and kerosene, mixed in equal proportions and applied to bedsteads, is an unfailing bug remedy, and that a coat of whitewash is ditto for a log house.

That beeswax and salt will make your rusty flat-irons as clean and as smooth as glass. Tie a lump of wax in a rag and keep it for that purpose. When the irons are hot, rub them first with the wax rag, then scour them with a paper or cloth sprinkled with salt.

